# Serial measurement of *M. tuberculosis* in blood from critically-ill patients with HIV-associated tuberculosis

**DOI:** 10.1016/j.ebiom.2022.103949

**Published:** 2022-03-21

**Authors:** David A. Barr, Charlotte Schutz, Avuyonke Balfour, Muki Shey, Mireille Kamariza, Carolyn R. Bertozzi, Timothy J. de Wet, Ryan Dinkele, Amy Ward, Kathryn A. Haigh, Jean-Paul Kanyik, Valerie Mizrahi, Mark P. Nicol, Robert J. Wilkinson, David G. Lalloo, Digby F. Warner, Graeme Meintjes, Gerry Davies

**Affiliations:** a Wellcome Centre for Infectious Diseases Research in Africa (CIDRI-Africa), Institute of Infectious Disease and Molecular Medicine, University of Cape Town, Observatory 7925, South Africa; b Institute of Infection and Global Health, University of Liverpool, Liverpool L7 3EA, UK; c SAMRC/NHLS/UCT Molecular Mycobacteriology Research Unit, Institute of Infectious Disease and Molecular Medicine and Department of Pathology, University of Cape Town, Observatory 7925, South Africa; d Department of Biology, Stanford University, Stanford, CA 94305, USA; e Department of Chemistry, Stanford University, Stanford, CA 94305, USA; f Howard Hughes Medical Institute, Stanford University, Stanford, CA 94305, USA; g Department of Medicine, University of Cape Town, Observatory 7925, South Africa; h The Francis Crick Institute, London NW1 1AT, UK; i Division of Infection and Immunity School of Biomedical Sciences, University of Western Australia, Perth, Australia,; j Division of Medical Microbiology, University of Cape Town, Cape Town, South Africa; k Department of Infectious Disease, Imperial College, London W12 0NN, United Kingdom; l Liverpool School of Tropical Medicine, Pembroke Place, Liverpool L3 5QA, UK; m Khayelitsha Hospital, Department of Medicine, Cape Town, South Africa

**Keywords:** HIV-associated tuberculosis, Pharmacodynamics, Sepsis, Blood stream infection, Critical-illness

## Abstract

**Background:**

Despite being highly prevalent in hospitalised patients with severe HIV-associated tuberculosis (TB) and sepsis, little is known about the mycobacteriology of *Mycobacterium tuberculosis* bloodstream infection (MTBBSI). We developed methods to serially measure bacillary load in blood and used these to characterise MTBBSI response to anti-TB therapy (ATT) and relationship with mortality.

**Methods:**

We established a microscopy method for direct visualisation of *M. tuberculosis* bacilli in blood using a novel lysis-concentration protocol and the fluorescent probe, 4-*N,N*-dimethylaminonaphthalimide-trehalose (DMN-Tre). We tested blood using GeneXpert® MTB/RIF-Ultra (Xpert-ultra) and Myco/F lytic culture after processing blood through lysis-wash steps to remove PCR inhibitors and anti-microbial drug carry-over. HIV-positive patients predicted to have MTBBSI gave blood samples 0, 4, 24, 48 and 72 h after ATT initiation. Bacillary loads were quantified using microscopy, Xpert-ultra cycle threshold, and culture time-to-positivity. Pharmacodynamics were modelled using these measures combined on an ordinal scale, including association with 12-week mortality.

**Findings:**

*M. tuberculosis* was detected in 27 of 28 recruited participants; 25 (89%) by blood Xpert-ultra, 22 (79%) by DMN-Tre microscopy, and 21 (75%) by Myco/F lytic blood culture. Eight (29%) participants died by 12-week follow-up. In a combined pharmacodynamic model, predicted probabilities of negative DMN-Tre microscopy, blood Xpert-ultra, or blood culture after 72 h treatment were 0·64, 0·27, and 0·94, respectively, in those who survived, compared with 0·23, 0·06, and 0·71 in those who died (posterior probability of slower clearance of MTBBSI in those that died >0·99). DMN-Tre microscopy of blood demonstrated heterogenous bacillary morphologies, including microcolonies and clumps. Bacillary cell-length varied significantly with ATT exposure (mean cell-length increase 0·13 log-µm/day; 95%CrI 0·10–0·16).

**Interpretation:**

Pharmacodynamics of MTBBSI treatment can be captured using DMN-Tre microscopy, blood Xpert-ultra and culture. This could facilitate interventional trials in severe HIV-associated TB.

**Funding:**

Wellcome Trust, NIH Fogarty International Center, South African MRC, NIHR(UK), National Research Foundation of South Africa.

**Research in context**

**Evidence before this study**

We searched PubMed using terms [("Blood stream infection" OR BSI OR bacter?emia OR septic?emia) AND (tuberculosis OR TB)] OR [mycobacter?emia] on 2021-12-01 without date or language restriction. *M. tuberculosis* bloodstream infection (MTBBSI) is present in 30–50% of adults admitted to hospital with HIV-associated tuberculosis (TB) and associated with 2-fold increased risk of death compared to *M. tuberculosis* blood culture negative disease. MTBBSI is also the leading cause of sepsis in studies from sub-Saharan Africa. MTBBSI and other markers of systemic dissemination have been associated with adverse host-response phenotypes by several groups.

In contrast to other high-mortality forms of TB such as TB meningitis, we found no dedicated interventional trials of management for MTBBSI. We found two small studies reporting quantification of MTBBSI using colony-forming unit counting, and no studies where serial quantification had been performed or where time to clearance of MTBBSI on treatment was observed, or where pharmacodynamics of MTBBSI treatment were assessed. We found zero studies where direct microscopy of blood was successfully used to visualise *M. tuberculosis* bacilli in blood.

**Added value of this study**

A novel 4-*N,N*-dimethylaminonaphthalimide-trehalose microscopy method allows TB bacilli to be directly visualised in patient blood samples. Using this technique, combined with blood Xpert-ultra cycle threshold and TB blood culture time-to-positivity, we report pharmacodynamics of MTBBSI for the first time. TB bacteraemia remains detectable in most patients after 72 h of ATT. Some patients with MTBBSI have remarkably high concentrations of bacilli in blood, which associates with clinical phenotype and markers of disease severity. Adverse dynamics of bacillary load in MTBBSI are associated with mortality in a pharmacodynamic model combining DMN-Tre microscopy bacillary counts, blood Xpert-ultra cycle threshold values, and Myco/F lytic blood culture time-to-positivity at different time points after initiation of ATT.

**Implications of all the available evidence**

Blood *M. tuberculosis* bacillary load can be serially measured in critically-ill patients with HIV-associated TB and is related to illness severity and risk of death. Serial measures of MTBBSI are a plausible pharmacodynamic biomarker giving *in vivo* read-outs of response to therapy. This motivates for interventional trials designed to optimise ATT in a patient population previously excluded from trials despite accounting for a high fraction of deaths in people living with HIV.

Alt-text: Unlabelled box

## Introduction

TB is the leading cause of hospital admission, sepsis, bacteremia, critical illness and death in people living with HIV.^1^, ^2^, ^3^, ^4^, ^5^, ^6^ Severe HIV-associated TB can be characterized by extensive bacillary dissemination, with associated inflammation, innate immune cell signaling, progressive organ dysfunction, and high risk of early mortality.^7^ *M. tuberculosis* bloodstream infection (MTBBSI) is frequent amongst critically-ill patients with HIV-associated TB, and a major independent predictor of mortality.^5^ A dose-response relationship exists between blood bacillary load, clinical and immunological phenotype, and risk of death.^7^^,^^8^

Apart from diagnostic trials,^9^ severe HIV-associated TB has not been the focus of dedicated interventional trials of therapies to reduce mortality. Adequacy of anti-TB treatment (ATT) in HIV-associated disease has been tested predominantly in smear-positive, ambulant outpatients with pulmonary TB, and not in critically-ill inpatients with MTBBSI.^10^ This is, at least in part, because ATT pharmacodynamic effects are traditionally assessed through serial quantification of *M. tuberculosis* in sputum. Such measurements have not previously been attempted in other body compartments, including blood.

Further, despite the importance of the bloodstream as a site of infection in severe HIV-associated TB, little is known about the mycobacteriology of MTBBSI, including the location of bacilli (*e.g.*, intra- or extra-cellular), their morphologies, and their physiological states. Direct visualization of bacterial morphologies under different antimicrobial exposures *in vivo* may allow inferences of pharmacodynamic responses at the single-cell level; for example, the cell elongation phenotype seen in a variety of bacterial pathogens in response to stress.^11^

We developed a method comprising three key innovations for measuring *M. tuberculosis* in blood during the first 72 h of ATT as potential pharmacodynamic biomarkers. Firstly, our lysis-concentration microscopy method uses the mycobacterial viability probe, 4-*N,N*-dimethylaminonaphthalimide-trehalose (DMN-Tre). DMN-Tre is metabolised by mycobacteria and other actinobacteria to a trehalose mycolate that is incorporated into the lipid-rich outer membrane, resulting in a >700-fold fluorescence increase.^12^ Consequently, DMN-Tre has specificity for actinobacteria (of which mycobacteria are the only major blood pathogens), and reports only viable, metabolically active organisms.^12^ Secondly, a lysis-wash method for removal of PCR inhibitors was developed, allowing blood to be tested on the GeneXpert® MTB/RIF Ultra platform (Xpert-ultra, Cepheid, Sunnyvale, California) with high sensitivity.^8^ Third, to allow culture of blood after initiation of ATT, we reduced antimicrobial carry-over by removing plasma or performing lysis-wash preprocessing of blood.

Applying these three innovations, *M. tuberculosis* was serially measured in blood over the first 72 h of TB therapy in a cohort of hospitalised patients with high predicted probability of MTBBSI. We describe the pharmacodynamics of ATT in patients with HIV-associated MTBBSI, and assess association with mortality. We also investigated *M. tuberculosis* cell morphology *in vivo* using the lysis-concentration DMN-Tre microscopy method, and assessed the relative bacilli load in different blood components using the lysis-wash Xpert-ultra method in a subset of samples.

## Methods

### Study design

A prospective cohort study was performed with serial measurement of *M. tuberculosis* in blood samples taken during the first 72 h of standard ATT between 11/08/2017 and 21/11/2017. Samples were collected at 5 timepoints: 0 h (prior to first dose of ATT, and 4, 24, 48, and 72 h after first dose of ATT. Vital status outcome at 12 weeks was ascertained by telephone call.

### Participants & setting

This study was nested within a larger cohort study^7^ set in Khayelitsha Hospital, Cape Town, a 240-bed hospital serving a population with high HIV seroprevalence and TB incidence. Recruitment to the present sub-study was enriched for patients with high predicted probability of MTBBSI using an ensemble of machine-learning models developed with data from the parent study, and wrapped in a web-based app’ available for use at the patient bedside [URL: https://davidadambarr.shinyapps.io/MTBBSI_v2/]. Adults (age >18 years) with known HIV infection and admitted to hospital with suspected new TB diagnosis within the last 24 h, with at least one suggestive clinical parameter (Fig. E2, online supplement), were formally screened and invited to participate if predicted MTBBSI probability was >0·6.

### Sample-processing

At each time point, 18 mL of blood was collected in sterile sodium heparin bottles; 5 mL was centrifuged at 3000 x *g* for 25 min and the cell pellet inoculated into a Myco/F Lytic culture bottle (BD Biosciences) reducing potential antimicrobial drug carry-over from plasma. The remaining blood was split into 10 mL and 3 mL aliquots. Both aliquots were mixed with 30 mL of 0.22 µm-filtered RBC lysis buffer [155 mM NH_4_Cl; 12 mM NaHCO_3_; 0.1 mM EDTA], incubated at room temperature for 30 min with inversions, then pelleted at 3000 x *g* for 25 min. This wash step was then repeated in 45 mL 0.22 µm-filtered deionised water. These wash steps were designed to remove free antimicrobial drugs (to facilitate culture), haem (a PCR inhibitor), and eukaryotic cell cells and debris (to facilitate downstream DMN-Tre microscopy).

Half the pellet from the 10 mL blood aliquot was inoculated into a second Myco/F Lytic bottle, and half was stored at -20 °C for downstream Xpert-ultra testing. The pellet from the 3 mL blood aliquot was resuspended in 5 mL of Myco/F Lytic broth containing DMN-Tre to a final concentration of 154 µM, and the sample incubated in the dark and under agitation for 18 h at 37 °C. After incubation, the sample was mixed with 25 mL of a detergent and enzymatic lysis buffer [0.22 µm-filtered 0.5% v/v Triton-X-100, 1.0% v/v Tween-80 deionised water with 0.8 mg/mL proteinase-K added] pre-warmed to 37 °C, and incubated at 37 °C for 25 min with inversions, before pelleting (3000 x *g*, 25 min) and resuspension in 2 mL 0.22 µm-filtered water. This lysis step was established empirically from permutations of published lysis methods and resulted in a filterable lysate without >10% loss of mycobacterial cells in spiked samples. The sample was then placed in a 13 mm sterile centrifuge ultrafiltration holder [UFREU1301, Sterlitech Corporation, Kent WA] pre-loaded with a 13 mm black 0.6 μm polycarbonate membrane filter [PCTB0613100, Sterlitech Corporation, Kent WA], which was then centrifuged at 1800 x *g* for 10 min. Filters were then sealed inside 20 mm glass bottom cell culture dishes [801001, Whitehead Scientific, Cape Town] mounted in an aluminium holder custom-made for the microscope stage.

Urine (50 mL) was collected daily, pelleted (3000 x *g*, 25 min), and stored at -20 °C for downstream Xpert-ultra testing.

### DMN-Tre microscopy

DMN-Tre microscopy was performed on a Zeiss Axio Observer 7 using the Plan-Apochromat 100x/1.4 Oil objective and 10x ocular lens giving 1000x magnification. Each membrane was examined for 2 h and, if bacilli were identified, 500 random fields-of-view (FOV) identified by a random array of stage coordinates were examined at 1000x magnification to quantify bacilli on the membrane. The effective surface area of the membrane was estimated to be 2850 FOV based on experiments with fluorescent beads counted by flow cytometry, giving bacillary count:

$$bacilli\;ml^{-1}=\;\frac{total\;microscopy\;count}{FOV\;examined}\;\times \frac{2850\;FOV}{3ml}$$

Bacillary counting was performed by one microscopist (DAB), with a random selection of 138 digital images captured using Carl Zeiss image software reviewed by a second analyst (KAH) to assess inter-rater agreement on classification of fluorescent foci as bacilli.

### Blood Xpert-ultra testing

Batched samples were thawed and tested using Xpert-Ultra cartridges.^8^ Full results were output from the Xpert software as .txt files and cycle threshold (Ct) values extracted using a custom R script. The mean Ct value of the four *rpoB* probes was used as the summary value; for *rpoB* probe negative / IS*1081*-IS*6110*-positive samples (“trace” positive), we used the IS*1081*-IS*6110* Ct values to impute mean *rpoB* probe Ct value.^8^

### Myco/F lytic blood cultures

Myco/F Lytic cultures inoculated as above were transferred to the National Health Laboratory Service (NHLS) and incubated within 24 h of venesection in the automated BACTEC system. Time-to-positivity (TTP) was extracted from BACTEC software.

### Separation of blood components in subset of samples

For 10 randomly selected patient-timepoints, a 5 mL blood sample was separated into plasma, a buffy coat layer containing poly- and mono-nuclear white blood cells, and red cell pellet using density gradient centrifugation (PolymorphPrep, PROGEN, Heidelberg, Germany), then each sub-component was processed for Xpert-Ultra testing using the same protocol as for whole blood. Ct values were pairwise compared across blood components to test if bacilli in MTBBSI were predominantly found in the buffy coat, as would be expected if they were intracellular within phagocytes.

### Statistical methods

To summarise participant characteristics and provide fundamental representations of clinical phenotypes potentially associated with blood bacillary load, dimension reduction methods were applied to clinical variables. Specifically, heatmaps of baseline clinical examination findings were hierarchically clustered using Euclidian distance and complete linkage (R *pheatmap* package), and also reduced to two dimensions of variation using Multiple Correspondence Analysis (MCA, using R *factoMineR* package). Blood results with continuous measures were similarly summarised using Principal Components Analysis (PCA, using R *psych* package). Association of MCA and PCA dimensions with measures of blood bacillary load at timepoint 0 were assessed graphically and by linear regression.

Blood result variables were chosen for inclusion based on availability and theorised representation of processes known to be important pathophysiology markers in severe HIV-associated TB or bloodstream sepsis: haemoglobin (hepcidin regulated anaemia of inflammation)^13^; platelet count and mean platelet volume (haemostatic dysregulation)^14^, ^15^, ^16^; total white cell and CD4+ cell count (inflammation and immunosuppression)^17^^,^^18^; urea, creatinine, and sodium (renal injury and anti-diuretic hormone secretion); residual variance in aspartate transaminase (AST) after adjusting for alanine transaminase (tissue damage / mitochondrial injury),^19^ lactate and glucose (inflammation induced accelerated aerobic glycolysis and sepsis).^20^^,^^21^

As a measure of co-validity, agreement and correlation between the three independent methods for identifying and quantifying MTBBSI, as well as urine Xpert-ultra results as a comparator, were assessed using Cohen's kappa, and by cubic spline regression for quantitative results.

We investigated the relationship between dynamics of blood bacillary load and mortality in a combined pharmacodynamic model as follows. The three quantitative measures of blood bacilli (DMN-Tre microscopy count, MFL culture TTP, and blood Xpert-ultra Ct) were each converted to an ordinal scale ranging from 1 for a negative test, to 10 for higher-load observed values, and regressed on variables: *time* in days from start of treatment, quantification *method* (DMN-Tre microscopy, MFL culture, or blood Xpert-ultra), and 12-week *outcome* (survived or died). Interactions between time and method, and time and outcome, were also included, allowing both intercepts and slopes to vary, to assess if overall bacilli load and/or rate of change in bacilli load on treatment varied across levels of these categorical variables. Random effects for intercept and slope by participant were used. The motivating assumption for this ordinal regression approach was that each of the three quantification methods could be treated as indicator variables reporting on an unobserved latent variable, namely blood bacilli load.^22^ It allowed all three measures to be represented on the same scale without making distributional assumptions (*i.e*., without assuming that the 3 measures were metric, with equivalent variances under different conditions assessed) and, further, allowed negative values to be included while accounting for non-normality from ‘ceiling’ and ‘floor’ effects, avoiding need for imputation of below limit-of-detection values.

As well as assessing blood bacilli load quantitatively, the DMN-Tre microscopy method allows assessment of treatment effects on bacilli at a single-cell level. We hypothesised that bacillary morphology, specifically cell length, might be affected by antimicrobial exposure *in vivo*. To evaluate cell-length distribution of bacilli, DMN-Tre microscopy digital images were captured and individual bacilli lengths measured with the segmented line function in Fiji ImageJ^23^ for 36 samples from the 10 patients with highest bacilli loads observed on DMN-Tre microscopy. The effect of exposure to antimicrobials, and heterogeneity in cell length between individuals, was tested with a linear mixed-effects regression modelling log bacilli length (assuming a log-normal distribution) as a function of *time* in days from start of treatment, with random-effects for intercept and slope by individual participant.

All regression models were fitted using a Bayesian approach, with parameters estimated using the No-U-Turn-Sampler algorithm implemented in Stan and compiled using the *brms* 2·13·5 package in R.^24^ Moderately informative priors were chosen for three beta coefficients. A normal (mean =1, SD= 0.5) prior for the mortality outcome beta coefficient was used, derived from blood Xpert-ultra *rpoB* Ct and MFL blood culture TTP data collected at a single timepoint (day of recruitment) in the larger parent study. For the *time* beta coefficient, a prior of normal(-0.5, SD=1) was set to reflect the prior belief that, on average, ATT exposure was more likely to decrease than increase bacillary numbers. For the bacillary cell-length model, a prior distribution for log cell-length in µM was set [normal(mean=0.43, SD=0.06)], derived from the work of Vijay et al. on *M. tuberculosis* measurements in patient sputum samples.^25^ Models were checked using graphical inspection of posterior distributions against observed data, trank plots, R-hat and effective sample estimates.

All analysis was performed in R version 3·5·2, with code and data available at https://github.com/davidadambarr/serial_mtbbsi .

### Ethics

Written informed consent was obtained from all participants; the study was approved by University of Cape Town Human Research Ethics Committee (ref 057/2013, 11 July 2017 amendment).

### Role of the funding source

The funders of the study had no role in study design, data collection, data analysis, data interpretation, or writing of the report.

## Results

Twenty-eight patients with high predicted probability of MTBBSI were recruited (Fig. E2, online supplement); 19 (68%) were female, median age was 35 years (IQR 30 to 40), median CD4+ cell count was 33 cells/mm^3^ (IQR 17 to 72), and 8 (29%) participants died by 12 weeks follow-up. Median time to death was 21 days (range 1–75 days). All participants started standard ATT (weight-based, 4‐drug fixed‐dose combination tablets containing rifampicin, isoniazid, ethambutol, pyrazinamide, as per national guidelines),^26^ except one patient treated with an isoniazid- and pyrazinamide-sparing regimen owing to abnormal liver biochemistry, and one patient treated without ethambutol owing to renal impairment. All participants had rifampicin drug-sensitive TB confirmed on direct sensitivity testing.

**Figure 1:**
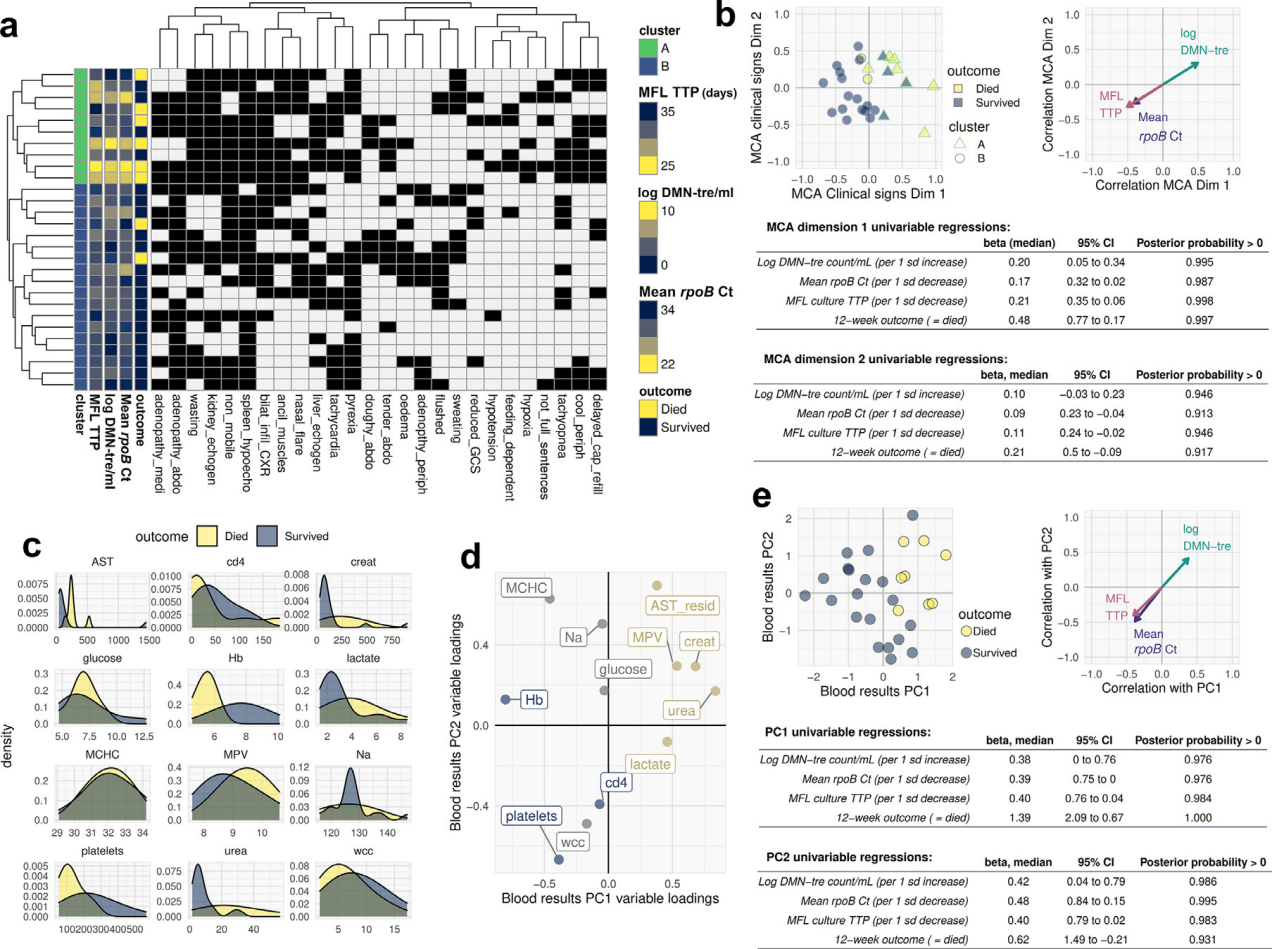
Description of clinical phenotype and relationship with outcome and markers of MTBBSI **a**. Heatmap of binary clinical signs from baseline assessment at time of recruitment (black tile = present, white tile = absent). Participants (rows) and clinical signs (columns) are hierarchically clustered (indicated by row dendrogram), with two highest level clusters of participants indicated by a colour key (cluster A, top ten rows, green; cluster B bottom 18 rows, blue). Each participant's 12-week outcome status and MTBBSI quantification results from day of recruitment are also shown on a navy-yellow colour scale to indicate level of correspondence with the clustered clinical signs data. The figure shows that patient clinical phenotype observed from the bedside, and classified by an unsupervised clustering algorithm, corresponds closely to blood bacillary load measures and risk of mortality. **b**. The same clinical signs data as above represented in two dimensions using Multiple Correspondence Analysis (MCA). Each point is a participant, with 12-week outcome status indicated by fill-colour, and cluster membership from panel A indicated by outline shape and colour. Also shown are projections of the markers of MTBBSI bacillary load in this MCA space: Pearson's correlation coefficient with MCA dimension 1 and 2 are indicated by arrowhead *x* and *y* coordinates respectively. Tables below show regression beta coefficients for univariable models regressing markers of MTBBSI bacillary load and 12-week outcome on MCA dimension 1 and 2, respectively. **c**. Density histograms showing distribution of 12 key blood results by 12-week outcome status. **d**. Principal Components Analysis with varimax rotation summarising the same 12 variables. Variables where higher observed values are associated with more adverse clinical status are coloured yellow; variables where lower values are broadly more adverse are coloured blue; and variables where derangement outside normal range in either direction is adverse are coloured grey. **e**. Projection of individuals and markers of MTBBSI bacillary load in the 2-dimensional blood assay PCA space, with univariable linear models regressing markers of MTBBSI bacillary load and 12-week outcome on PC 1 and 2 respectively. Hb = haemoglobin; MCHC = mean corpuscular haemoglobin concentration; platelets = platelet count x10^9^/L; MPV = mean platelet volume; wcc = total white cell count x10^9^/L; cd4 = CD4+ cell count cells/mm^3^; urea = serum urea mmol/L, creat = serum creatinine umol/L; Na = serum sodium mmol/L; AST = aspartate transaminase U/L; lactate = venous lactate mmol/L; glucose = venous glucose mmol/L. AST_resid = residual variance in aspartate transaminase after adjusting for alanine transaminase.

As expected from the selection criteria, participants were seriously ill with high prevalence of organ dysfunction and adverse clinical markers (Figure 1), including cool peripheries, tachypnoea, use of accessory muscles of breathing, functional impairment, anaemia, kidney injury, AST elevation independent of variance in ALT, and thrombocytopenia with increased mean platelet volume (implying platelet destruction rather than bone marrow failure). Even within this seriously-ill patient stratum, the major components of variation in clinical phenotype correlated with measures of MTBBSI bacillary load and outcome (posterior probabilities of non-zero effect size > 0.9, Figure 1).

**Figure 2:**
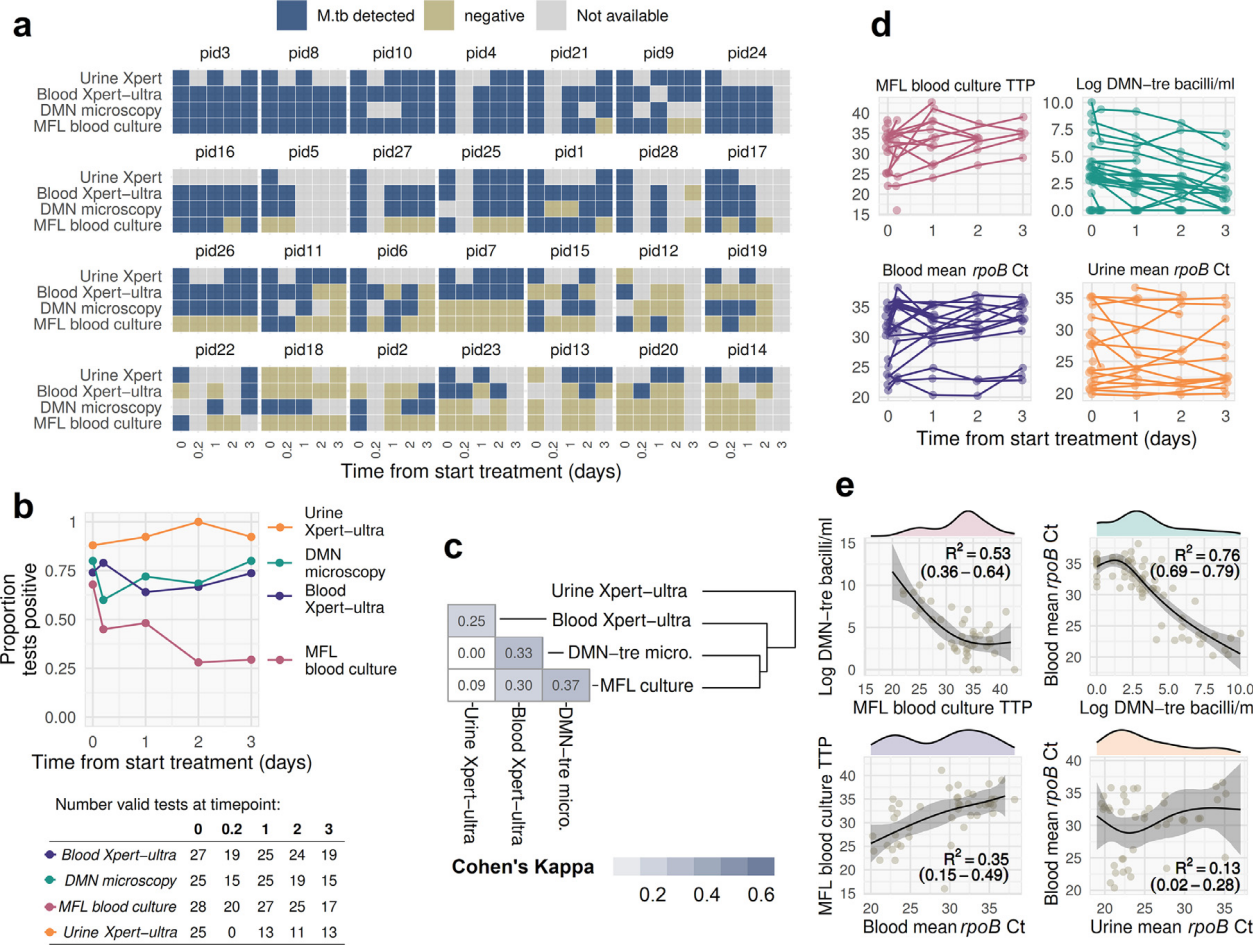
Results of serial quantification of MTBBSI during first 3 days of anti-TB therapy Qualitative and quantitative results of three MTBBSI detection methods, with urine Xpert-ultra results as a comparator. **a**. Time plot showing qualitative results by patient timepoint. pid = Patient identity number from study. Data from 20 patient time-points are missing because either patient had died (*n* = 3), or was unavailable/declined venesection (*n* = 17). Remaining non-available samples are from technical failures including contamination and lost samples. **b**. Proportion of positive test results by time. Valid available tests were used as denominator, as shown in “at risk” table below plot. **c**. Pairwise agreement between test results assessed by Cohen's Kappa statistic, with hierarchical clustering applied to the pairwise Kappa values shown with dendrogram. **d**. Quantitative results by timepoint. Lines connect data from individual participants. Negative samples are not plotted for Xpert-ultra and MFL culture; negative DMN-Tre microscopy results plotted as 0 values. **e**. Univariate (density histogram for x-axis variable) and bivariate (scatter plot) distributions of quantitative results from (D). Bivariable natural cubic spline regression with three degrees of freedom also shown: black line indicates median posterior fitted values; shaded band is 95% interval for posterior fitted values. As assessed by leave-one-out cross-validation based on the posterior likelihood, non-linear cubic spline models had significantly better fit than corresponding linear models for all the observed bivariable distributions, with the exception of blood ∼ urine mean *rpoB* Ct which is included as a comparator only.

Results for at least two of the three MTBBSI assays were available for 116 (83%) of the 5 timepoints in 28 patients (140 patient-timepoints). MTBBSI was detected in at least one sample for 27 (96%) participants. Overall, of 114 available blood Xpert-ultra test results, 81 (72%) were positive; of 99 available DMN-Tre microscopy results, 72 (72%) showed bacilli; and of 117 timepoints with available MFL blood culture result, 53 (45%) had ≥1 bottle recovering *M. tuberculosis* (Figure 2A). Inter-rater agreement for DMN-Tre microscopy digital images was high (Cohen's Kappa 0·86, *p* < 0·0001). The proportion of positive test results remained around 75% for blood Xpert-ultra and DMN-Tre microscopy after 72 h of ATT, while MFL blood culture positivity declined from 68% at baseline to 29% at 72 h (Figure 2B). MTBBSI bacillary load declined over time in most patients irrespective of quantification method (Figure 2D). All three MTBBSI quantifications showed strong pairwise correlation, but with evidence of non-linearity (Figure 2E).

**Fig. 3:**
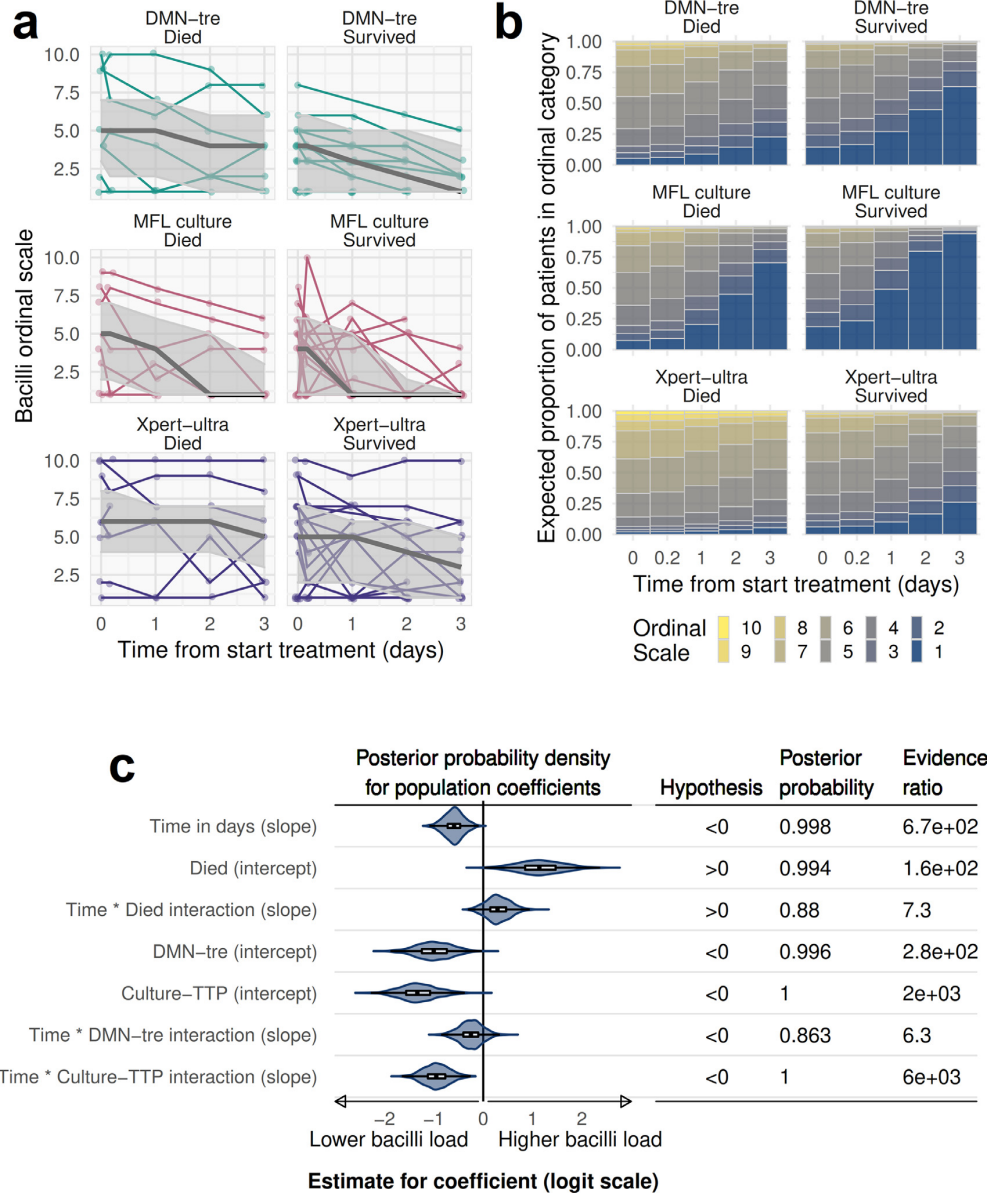
Ordinal regression modelling pharmacodynamics of MTBBSI bacillary load and relationship with mortality **a**. Observed values for MTBBSI quantification results on ordinal scale by timepoint, disaggregated by method and outcome status, are shown with coloured lines connecting individual participants’ results. Overlaid on this raw data are predicted values from the ordinal regression model by the three fixed-effect (population-level) predictor variables: timepoint, method and outcome status. Black line indicates median predicted value from 1000 draws from the model posterior predictions; shaded area is the 50% prediction interval (interquartile range for 1000 draws from the model posterior predictions). This prediction interval includes uncertainty in location of the population-level parameters (intercepts and beta coefficients) as well as residual variation and random effects for individual participant; the 50% prediction interval therefore represents simulated values for new unobserved patients drawn from the patient population under study. **b**. Expected proportions of patients in each ordinal scale category by timepoint, method and outcome status based on model fit. These are mean posterior probabilities for each ordinal category, for an “average” participant (*i.e*., with random-effects of 0). **c**. Uncertainty in model fixed-effect (population-level) beta coefficients. Posterior distribution of coefficients are shown with violin plots and box-plots (range, interquartile range and median), on logit scale. For example, median value for the *Time in days (slope)* coefficient is -0.58; this indicates an odds ratio of exp(-0.58) = 0.56 for difference in bacillary load ordinal distribution toward category 10, per 1-day increase in time since start of ATT; *i.e*., lower ordinal categories with time. Patients who died had on average greater odds of higher ordinal category distribution compared to those who survived (*Died (intercept)* coefficient), and a less rapid shift in ordinal distribution towards category 1 over time on ATT (Time * Died interaction (slope) coefficient). DMN-Tre and MFL blood culture ordinal scale distributions on average had lower odds of higher ordinal distribution compared to blood Xpert-ultra. Evidence that coefficients were different from 0 (null effect) in direction indicated is formally tabulated: posterior probability shows proportion of posterior distribution of coefficient estimates > or < 0 as indicated; evidence ratio is the evidence of posterior distributions for and against the stated hypothesis. For example, the model estimates a probability of 99.8% that *Time in days (slope)* coefficient is less than 0; *i.e*., that ordinal distribution shifts towards category 1 with time, and 88% probability that this shift is less marked in those that died.

All three MTBBSI assays were scaled into ordinal categories ranging from 1 (negative sample) to 10 (corresponding to highest observed DMN-Tre bacillary count, lowest blood Xpert-ultra Ct and MFL culture TTP values) and jointly modelled in an ordinal regression analysis summarising pharmacodynamics of MTBBSI over the first 3 days of ATT (Figure 3). From this model, there was strong evidence that, on average, MTBBSI bacillary load declined over the first 3 days of therapy: the probability of higher ordinal category bacilli load reduced with time on treatment (odds ratio for higher ordinal distribution per 1 day increase in time since starting therapy 0·56, 95% credibility interval 0·38 to 0·82, with 0·998 posterior probability that this odds ratio was below 1). Participants who died had greater probability of higher bacillary load ordinal distribution at all timepoints compared to those who survived (OR 3·1; 95%CrI 1·2 to 7·7; probability OR >1 = 0·994), and some evidence that the shift to lower bacillary load ordinal distribution occurred more slowly in those who died (OR for interaction between time on treatment and died outcome 1·3; 95%CrI 0·8 to 2·2; probability OR >1 = 0·880) (Figure 3C). For “average” patients (zero random effects), predicted probabilities of negative DMN-Tre microscopy, blood Xpert-ultra, or MFL culture after 3 days of treatment were 0·64, 0·27, and 0·94, respectively, in those who survived, compared to 0·23, 0·06, and 0·71 in those who died (Figure 3B). Estimated median time in days until 50% probability of a negative DMN-Tre microscopy, blood Xpert-ultra, or MFL culture sample extrapolated from the model was 2·3 (95%CrI, 1·8 to 2·8), 4·8 (95%CrI, 4·0 to 6·1), and 1·0 (95%CrI, 0·8 to 1·2), respectively, in those who survived, compared to 5·5 (95%CrI, 4·2 to 7·6), 11·5 (95%CrI, 8·3 to 18·4), and 2·2 (95%CrI, 1·8 to 2·6) in those who died.

**Figure 4:**
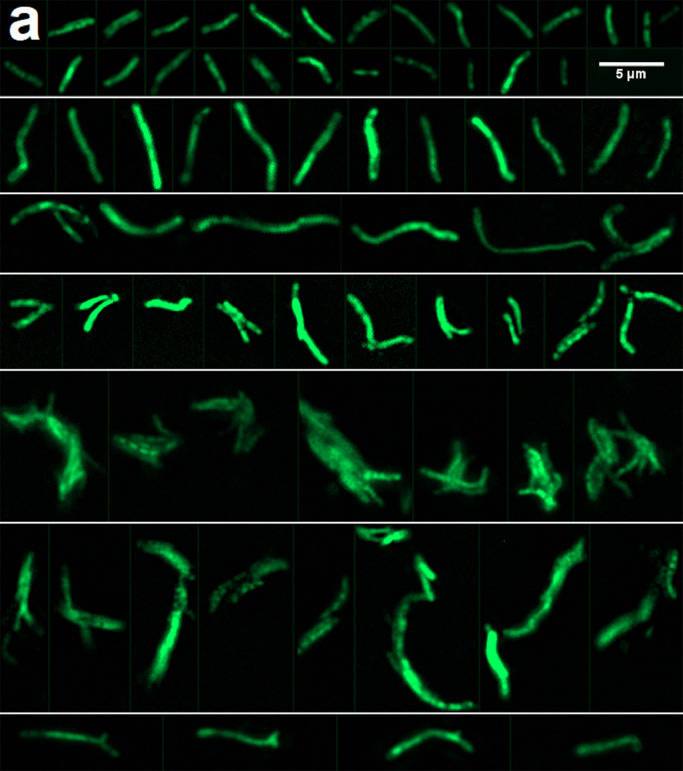

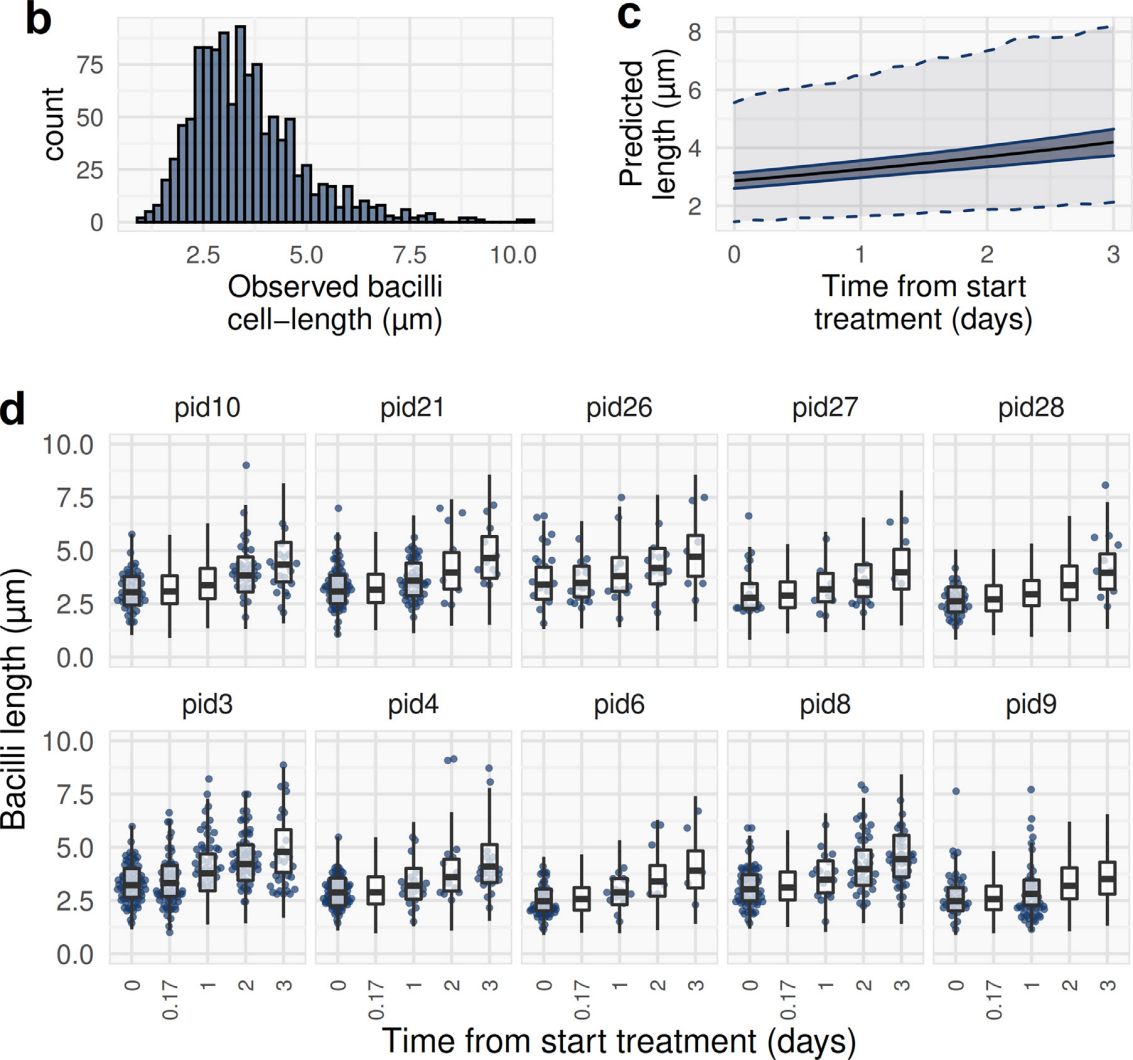
Microscopy analysis of bacillary morphologies in blood using DMN-Tre fluorescence **a**. DMN-Trehalose microscopy of MTBBSI bacilli. Shown are images from several patients, which have been ordered by bacillary morphological characteristics not by patient. Most bacilli were 2–4 µm long curved rods (top row panel), but cell-lengths 4–6 µm (second row panel) and longer (third row panel) were observed. Homogenous and heterogenous DMN-Tre probe uptake is shown. Doublets were seen (fourth row panel). Microcolonies and clumps were observed in some participants (fifth and sixth row panels). Morphologies suggestive of branching were also occasionally observed (seventh row panel). **b**. Distribution of measured cell-lengths for 1200 bacilli in blood samples from 10 participants. Bin widths are 0.2 µm. **c**. Expected and predicted cell-length in µm from mixed-effects regression of log cell-length on time in days, with random intercept and slope by participant. Median and 95 % credible interval for median cell-length are shown (indicating population-level fixed effect of time); outer dashed lines show 95 % prediction interval where 95 % of bacillary lengths are predicted to be in any given patient (indicating random variation between patients plus residual variation). **d**. Model predicted posterior distribution (boxplots) overlaid on observed cell-length values by participant and timepoint demonstrating model fit to data.

Twenty-two participants had at least one DMN-Tre microscopy positive blood sample. Maximum observed bacillary count by participant ranged from 10 to 23,000 bacilli/mL. DMN-Tre microscopy revealed heterogeneity in the morphologies of bacilli recovered from blood (Figure 4A). Microcolonies and clumped cells were seen in multiple samples from 7 patients. Cell-lengths were measured on images of 1200 bacilli from the 10 participants with highest bacillary loads. Most bacilli were 2–4 µm long curved rods, but longer bacilli up to approximately 10 µm were seen in a log-normal distribution of cell-lengths (Figure 4B). A mixed-effects model of this data demonstrated significant inter-patient heterogeneity in blood bacillary cell-lengths (between participant sd 0·13 log-µm; 95%CrI 0·07 to 0·24), and significant increase in mean bacillary length with exposure to ATT (mean cell-length increased by 0·13 log-µm per day of ATT; 95%CrI 0·10 to 0·16; posterior probability for increasing cell-length by timepoint >0·999, Figure 4C&D).

Paired Xpert-ultra Ct values were lower but similar for whole blood compared to the ‘buffy coat’ white blood cell fraction (posterior probability that whole blood Ct values were on average lower than buffy coat Ct values was 69%); whole blood Ct values were more convincingly lower on average than red-cell pellet Ct values, while plasma Ct values were highest in all cases (Fig. E3 online supplement). Overall, the rank order for Ct values of [buffy coat < red-cell pellet < plasma] had 93% posterior probability compared to any other permutation.

## Discussion

Bacteraemic *M. tuberculosis* can be detected and measured over the first 72 h of ATT using a novel quantitative DMN-Tre microscopy method, culture time-to-positivity and Xpert-ultra testing of blood pre-processed using the methods described. Time to clearance of MTBBSI in response to ATT was significantly slower in patients who died during the first 12 weeks of treatment. The serial quantitative read-outs of MTBBSI give robust prognostic information and should therefore be considered potential pharmacodynamic biomarkers in severe HIV-associated TB.

Hospitalised patients with severe HIV-associated TB have been excluded from ATT efficacy studies, so the adequacy of standard ATT is not well established for this group. The exceptionally high risk of death in this patient population – 12-week mortality is 30% in those with MTBBSI^5^^,^^7^ – suggests that optimising treatment efficacy is important. Pharmacodynamic biomarkers offer greater statistical power than dichotomous outcomes such as mortality, increasing efficiency of phase II trials and therefore potentially accelerating development of evidence-based therapies. Correlation of a biomarker with clinical outcome at the individual level does not ensure correlation at group (or trial arm) level.^27^ Biological plausibility that a biomarker lies on the *causal* pathway between intervention and outcome is therefore desirable, and bacteriological read-outs have a strong *a priori* case for meeting this condition.^28^ In support of a causal relationship, we found that blood bacillary load measured by the novel methods described had a dose-response relationship with clinical phenotype and mortality, adding to previous evidence.[8]

Two attempts at quantifying MTBBSI have been previously reported, both using lysis-centrifugation colony-forming unit (CFU) counting on pre-treatment blood samples.^29^^,^^30^ Crump et al. found <1 to 150 CFU/mL (median ∼40) in 9 patient blood samples; Munseri et al. found 1 to 23 CFU/mL (median ∼3) in 21 patient blood samples. Direct microscopy to detect *M. tuberculosis* in blood was attempted in the early 20th Century but subsequently abandoned owing to technical difficulties.^31^ This study reports the first successful use of microscopy on blood, with bacilli identified in 68 out of 99 total patient samples examined. Maximum observed DMN-Tre microscopy count by participant had a right-skewed distribution up to 23,000 bacilli/mL. We also found heterogenous bacillary morphologies, including evidence of microcolonies in patient blood samples, consistent with active growth.^32^ Bacteraemia densities greater than 10,000 bacilli/mL are not typical of other pathogens,^33^^,^^34^ but have been reported for *M. avium* complex.^35^

We found bacillary cell-length in blood to vary in a log-normal distribution, median 3 µm and range 1·2–10 µm. This matches reported cell-length distributions for bacilli in sputum, bronchial lavage and bioaerosols in patients with pulmonary TB,^25^^,^^36^^,^^37^ but not in lung cavities.^36^ We show significant heterogeneity in bacillary cell-length in blood between patients, which also replicates reports for *M. tuberculosis* in sputum where bacillary elongation has been associated with host stresses.^25^ In addition we show a significant increase in cell-length over the observed 3 days of ATT. The apparent elongation of *M. tuberculosis* bacilli in response to antimicrobial-stress is tentatively suggestive of filamentation – growth without division – which has been reported for other bacteria.^11^ Taken together, our results suggest that DMN-Tre microscopy of blood is an informative and novel tool providing single-cell phenotypic information relevant to patho-biology of severe HIV-associated TB – in particular, the mycobacterial response to antimicrobial exposure *in vivo*.

Dissemination of bacilli via blood was considered a fundamental step in the natural history of TB in classical descriptions of infection from the pre-antimicrobial era.^38^, ^39^, ^40^ This is supported by contemporary pathophysiology studies.^41^^,^^42^ However, how bacilli access and disseminate in blood remains unclear.^43^ In the zebrafish model, macrophages infected with mycobacteria egress from granulomas, translocating the endothelium to disseminate their intracellular bacilli via the bloodstream.^44^ Infected macrophages may further facilitate this by stimulating angiogenesis at granuloma sites through VEGF secretion.^45^ *M. tuberculosis* is detected in peripheral blood mononuclear cells during active and latent infection,^42^^,^^46^^,^^47^ again implying intracellular dissemination. Alternatively, free bacilli have been shown to adhere to and translocate through epithelial and endothelial cells *in vitro*.^48^ The interaction of mycobacteria with host epithelial cells is dependent on the heparin-binding haemagglutinin adhesin, loss of which prevents this haematogenous dissemination of extracellular bacilli in mice.^49^ We detected *M. tuberculosis* in all blood components tested, and Ct values were similar in paired buffy-coat and red-cell pellet samples. This implies that, in patients with advanced HIV-associated TB, bacilli in the bloodstream are not exclusively within phagocytic cells but are able to disseminate extracellularly or in association with erythrocytes.

Limitations of this study include the single-centre recruitment and small sample size with resulting uncertainty around key parameters. More replicates would have added power to detect differences, but we were limited by what was regarded as a safe volume for venesection. Because most patients remained blood Xpert-ultra and DMN-Tre microscopy positive at the last observed timepoint, median time-to-sterilisation of blood by these assays could only be extrapolated; in future, later timepoints should be assessed. The use of quantitative data for all three assays in one ordinal regression model should, however, increase confidence in the overall trends. Although the DMN-Tre microscopy method offers significant advances in characterising MTBBSI and relative specificity for mycobacteria, it is labour intensive and – as with all manual microscopy – requires subjective calls when classifying fluorescent objects as bacilli. Of note, all patients who were DMN-Tre positive had *M. tuberculosis* confirmed by reference laboratory culture from at least one sample, reducing risk of false positive results. Future goals should include automation and high throughput adaptions of the DMN-Tre microscopy. Finally, while we provide the first evidence that tracking bacilli load in blood is a potential pharmacodynamic metric, such biomarkers can only be validated in interventional trials.

## Contributors

Study conception and design: DAB, GM, GD, CS, DGL. Clinical data acquisition: DAB, CS, AW, J-PK. Development laboratory methods: DAB, MS, MK, CRB, TdW, RD, VM, DFW. Laboratory methods oversight: GM, GD, DFW, VM, MPN, RJW, CRB. Clinical oversight: CS, GM, GD, DGL. Laboratory data acquisition: DAB, MS, AB. DMN-Tre invention and production: MK, CRB. Image interpretation and processing: DAB, KAH, TdW, RD. Provision laboratory facilities and storage: GM, RJW, DFW. Data analysis: DAB with oversight from GD, GM, DFW. DAB wrote the manuscript with review and contribution from all co-authors. All authors read and approved the final manuscript. DAB & GD verified underlying data.

### Data sharing statement

All code and data available at https://github.com/davidadambarr/serial_mtbbsi.

## Declaration of interests

CRB and MK, are cofounders of OliLux Biosciences which has licensed patents related to the Two dyes presented in this paper. All other authors have declared that no conflict of interest exists.

## Appendix Supplementary materials

Image, application 1
