# Supplementary material for: Serial measurement of M. tuberculosis in blood from critically-ill patients with HIV-associated tuberculosis
Source: eBioMedicine. 2022 Mar 21;78:103949. doi: 10.1016/j.ebiom.2022.103949 (PMC8938880; doi:10.1016/j.ebiom.2022.103949)
Supplement: Supplementary file 1 [file mmc1.docx]

**Supplemental figures**

Figure E1. **Sampling schedule and processing summary.**


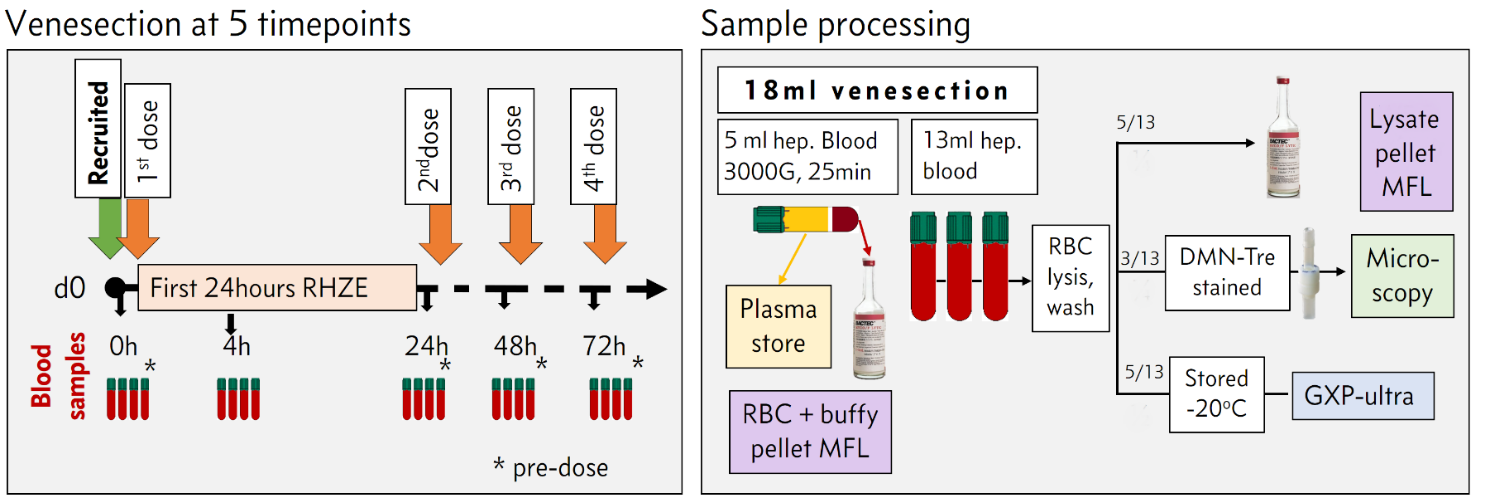


**Figure E1 legend:**

Dose refers to anti-TB therapy dose; hep. = heparinised; RBC = red blood cell; buffy = buffy coat; MFL = Myco/F lytic blood culture bottle; GXP-ultra = GeneXpert® MTB/RIF Ultra; 3000G, 25min = centrifugation at 3000 x *g* for 25 minutes.

Figure E2. **Participant screening and recruitment flow diagram.**


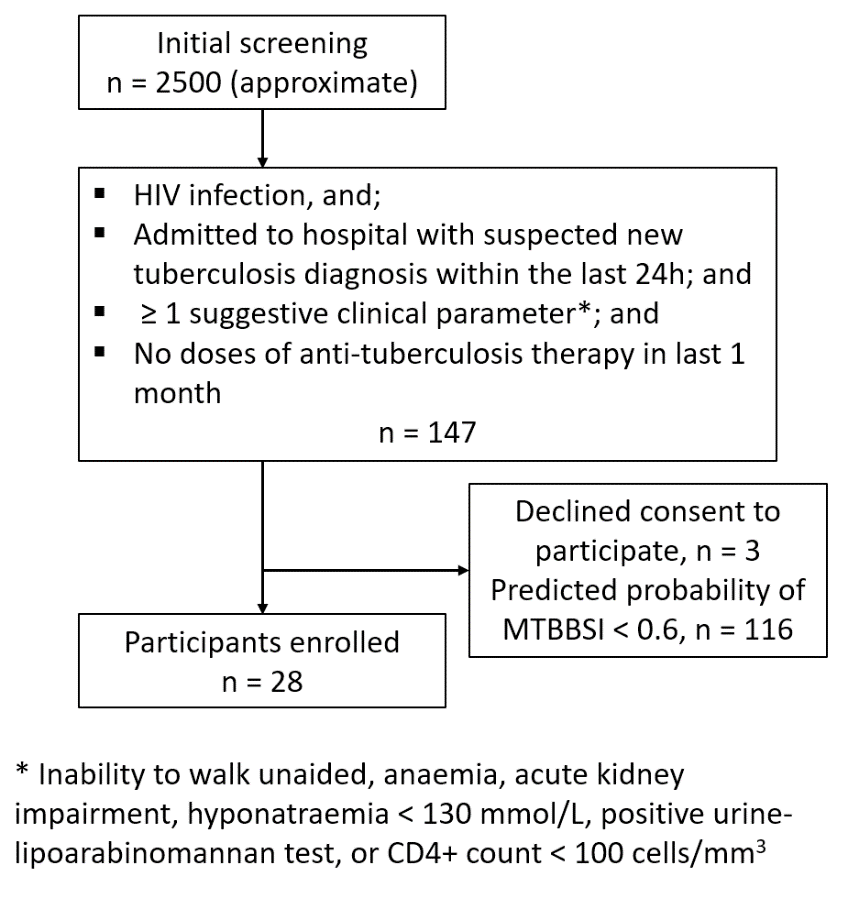


**Figure E2 legend**:

Inability to walk unaided, anaemia, acute kidney impairment, hyponatraemia < 130 mmol/L, positive urine-lipoarabinomannan test, or CD4+ count < 100 cells/mm^3^

Figure E3. **Relative quantification of *M. tuberculosis* in different blood components in paired samples**


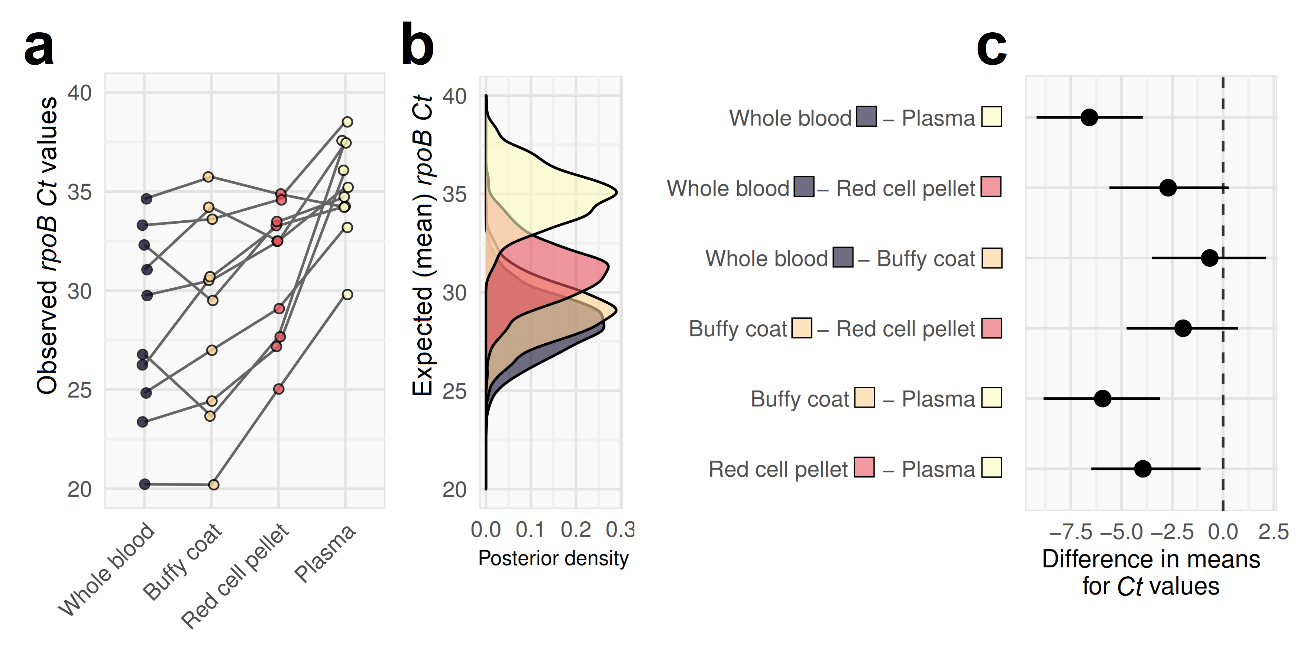


**Figure E3 legend**:

n = 10 paired samples (same patient-timepoint) were available to compare Xpert-ultra Ct values (summarised as mean across 4 probes, “trace” positive samples imputed as described in methods). One sample of the pair was processed as whole blood using the standard SOP; the other sample was split using density gradient centrifugation into plasma, buffy coat (a white cell pellet including mononuclear and polymorphonuclear cells), and the remaining red cell pellet. **a**. Observed Ct values for whole blood and the 3 sub-components are shown, with values from same sample-pair connected by a line. **b**. This data was modelled with fixed-effect for blood component and a random-effect for patient-timepoint sample pair. The posterior distribution for expected (*i.e*., mean) value by blood component is shown from 1000 draws from this model. **c**. Differences in these mean values (median difference and 95% credible interval) are shown for each blood component comparison.
